# Trends and perspectives in deterministic MINLP optimization for integrated planning, scheduling, control, and design of chemical processes

**DOI:** 10.1515/revce-2024-0064

**Published:** 2025-03-26

**Authors:** David A. Liñán, Luis A. Ricardez-Sandoval

**Affiliations:** Department of Chemical Engineering, University of Waterloo, 200 University Ave W, Waterloo, ON, Canada

**Keywords:** design, scheduling, control, planning, optimization; process integration

## Abstract

Mixed integer nonlinear programming (MINLP) in chemical engineering originated as a tool for solving optimal process synthesis and design problems. Since then, the application of MINLP has expanded to encompass control and operational decisions that are in line with the arising challenges faced by the industry, e.g., sustainability, competitive markets, and volatile supply chain environments. Nowadays, process plants are transitioning from traditional manufacturing practices to automated solutions able to integrate decision-making within manufacturing enterprises. This paradigm shift aims to increase profits, optimize resource utilization efficiency, promote long-term sustainability, minimize waste, and enhance responsiveness under uncertainties and perturbations. Accordingly, the development of reliable, computationally efficient, and robust MINLP algorithms capable of simultaneously handling process design, planning, scheduling, or control decisions are crucial to achieving Industry 4.0 integration goals. This work explores potential research opportunities and recent advances toward the development of integrated decision-making frameworks, focusing on their underlying state-of-the-art optimization tools. We provide an overview of emerging deterministic MINLP optimization algorithms for simultaneous decision-making problems. Furthermore, we constructively discuss the versatility and limitations of these optimization tools. We also highlight how novel optimization theories, both within and outside the chemical engineering domain, can be incorporated into advanced MINLP frameworks suitable for process integration.

## 1 Introduction

Digital transformation tools are progressively becoming relevant to increase the efficiency of production processes in the current context of a competitive industrial production environment (Csalódi et al. 2021). In this domain, the adoption of effective decision-making strategies plays a significant role to meet the economic, environmental, and safety standards imposed by the chemical, energy, pharmaceutical, and food industries. The different layers that define the decision-making process in the industry are traditionally classified using a hierarchical structure. The operational decisions that occur at different time scales can be generally categorized into planning (e.g., months-weeks), scheduling (e.g., days-hours), and the different layers associated with process control (e.g., minutes-seconds). At the core of the layers lies the actual design of the process. It has been reported that the lack of integration between these layers has resulted in suboptimal and even infeasible operation strategies (Rafiei and Ricardez-Sandoval 2020). This has motivated the development of integrated decision-making techniques that encompass at least two layers of integration from planning, scheduling, control, or design decisions. Nonetheless, the development and deployment of tools for optimal decision-making still poses many multidisciplinary research challenges in different domains including, but not limited to (Pistikopoulos et al. 2024):

1) Mathematical modeling (e.g., first-principles and data driven) and model reduction techniques,
2) Optimization algorithms able to provide reliable and timely solutions,
3) Closed-loop strategies that adequately use feedback from uncertain environments to handle set-point changes and disturbances,
4) Strategies to effectively handle large volumes of historical/current data coming from different sources in complex real-world systems,
5) Continuous synchronization between the real system and predictions coming from models in a simulated environment,
6) Changes in organizational and human behavior to implement new decision-making tools, and
7) Development of commercially available software prototypes for optimal process integration.

**Figure 1:**
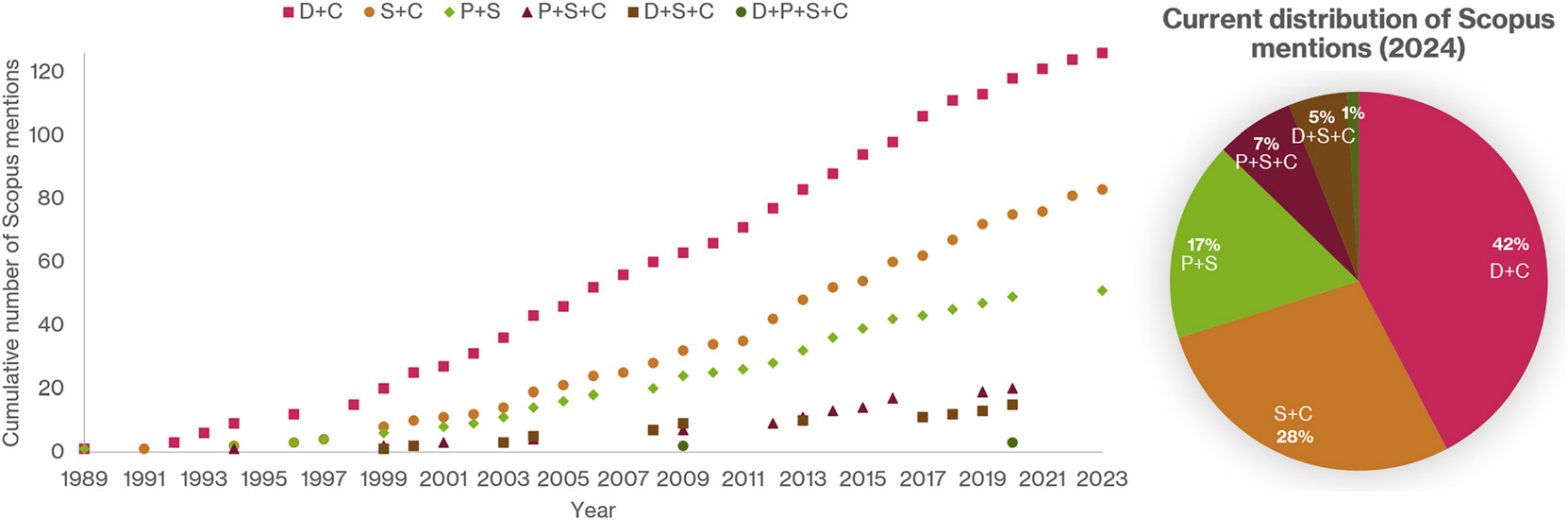
Distribution of works in the area of MINLP for integrated decision-making in chemical engineering. P: planning, S: scheduling, C: control, D: Design. The “+” symbol denotes the integration between layers, e.g., D + C means design and control.

Despite the equally important relevance of the challenges listed above, this review focuses on the second item listed above, i.e., optimization algorithms. Optimal integrated decision-making, in the context of chemical, energy, pharmaceutical, and food production processes, introduces two fundamental limiting factors related to the core idea behind plantwide integrated decision systems, namely the optimal solution of a high-fidelity representation of real-world phenomena (Biegler and Grossmann 2004). The first limiting factor is the presence of nonlinearities, which usually appear in the design and control layers (e.g., nonideal thermodynamic models and reaction rate expressions emerging in dynamic models), but they may also appear in the planning or scheduling layers (e.g., when considering simultaneous scheduling and blending). The second limiting factor is the presence of discrete variables in the formulation, most commonly in the process design layer (e.g., number of processing units in a reaction or distillation network), planning (e.g., number of wells for offshore oil and gas planning), scheduling (e.g., sequencing of tasks within a time horizon) layers, and sometimes in the control layer, e.g., hybrid MPC (Oberdieck and Pistikopoulos 2015). Consequently, integrated decision-making formulations can be generally classified as mixed integer dynamic optimization (MIDO) problems or Mixed Integer Nonlinear Programming (MINLP) problems once the differential equations involved are discretized using available numerical methods, e.g., orthogonal collocation (Biegler 1984). As indicated in previous studies, the solution of these problems is not a trivial task. Figure 1 shows the Scopus mentions to date (2024) of MINLP and MIDO problems in design, planning, scheduling, control, and typical examples of their integration in chemical engineering. According to Figure 1, the integration of two decision layers, particularly in the domains of scheduling and control (S + C) and design and control (D + C), has been the main research topic of interest within the PSE community.

Existing reviews and perspectives in the field of integrated design, planning, scheduling, and/or control aim to cover many of the integration challenges listed above, but they often fail to thoroughly explore in depth each domain, leading to a shallow look at the complex aspects of deterministic MINLP optimization for integrated decision-making, e.g., see (Andrés-Martínez and Ricardez-Sandoval 2022b; Burnak et al. 2019a; Pistikopoulos et al. 2015, 2024). Furthermore, recent reviews in the broader MINLP area do not have a focus on optimal process integration, e.g., see (Boukouvala et al. 2016; Kronqvist et al. 2019; Trespalacios and Grossmann 2014); hence, future research directions on the application of recently developed MINLP algorithms in the field of process integration have not been discussed in the literature. To fill these literature gaps, the first objective in this work is to cover the extensive progress that has been made in the development of MINLP optimization algorithms capable of handling large-scale and complex problems that arise in integrated decision-making. Although the focus is on MINLP techniques specifically tailored for decision-making integration, we briefly highlight the relevance of general-purpose MINLP solvers, i.e., those algorithms that are readily available through algebraic modeling languages (e.g., DICOPT, SBB, BARON, etc.). The second objective is to provide an overview of the extensive collection of MINLP algorithms that have been proposed in recent years and highlight their potential benefit in addressing integrated decision-making problems. While the present work focuses on model-based deterministic MINLP optimization algorithms for integrated decision-making, we also acknowledge the importance of related and relevant fields that are not covered in this review, e.g., black-box, derivative-free and heuristic methods (Ji and Gu 2021; Ramírez-Márquez et al. 2023); artificial intelligence advances to aid decision-making such as reinforcement learning and machine learning techniques (Gao and Schweidtmann 2024; Schweidtmann et al. 2021); controllability index approaches (Yuan et al. 2012); advances in convex relaxations and generalized derivatives for global MINLP optimization (Barton et al. 2018; Sahinidis 1996; Song et al. 2021; Tawarmalani 2002), purely data-driven MINLP methods where a first principles model of the system is not available (Davis and Ierapetritou 2008; Kim and Boukouvala 2020; Morlet-Espinosa and Flores-Tlacuahuac 2024), among others.

For completeness, this review begins by providing illustrative examples that explain the concept of integrated decision-making and its relevance to chemical engineering. Then, this work discusses the importance and limitations of general-purpose MINLP solvers when solving integrated decision-making problems. This is followed by an overview of the most widely used strategies to solve MINLP integrated decision-making problems, other than the direct use of general-purpose solvers. We then present a review of the latest MINLP algorithmic developments and explain how these methods can facilitate the solution of integrated decision-making problems in chemical engineering. Final remarks are provided at the end.

## 2 MINLP decision-making examples

This section highlights the importance of the integrated MINLP decision-making problems through illustrative examples in the fields of simultaneous design and control (D + C) scheduling and control (S + C), and planning and scheduling (P + S).

D + C problems typically optimize an economic objective function (e.g., sum of capital and production costs). The optimization may seek the optimal combination and interconnections within a set of available unit operations (e.g., select and interconnect different reactor, distillation column, and heat exchanger alternatives) and/or the optimal steady state operating conditions and discrete and continuous design parameters of each unit operation (e.g., reactor and distillation sizing). These problems include control considerations in the constraints through closed-loop or open loop dynamic models and/or the objective function through controllability/feasibility/stability measures (e.g., the integral squared error of a controlled variable with respect to its set point). Thus, simultaneously combining D + C results in an economic design with improved dynamic responsiveness under disturbances, uncertainties, or set-point changes. Similarly, simultaneous S + C aims to incorporate the closed-loop/open-loop dynamic models of unit operations into the scheduling problem, which aims to find the optimal start times, durations, and processed volumes of different tasks within a time horizon with respect to an objective function that is usually economic. S + C thus allows the generation of economic production plans that comply with the dynamic operation capabilities of the unit operations involved in the process. P + S incorporate planning decisions that do not occur at plant level, but at a business management level and coordinate the operation over a longer time scale. Planning decisions include strategic decisions such as capital expenditures, contracts, acquisitions, as well as tactical decisions related to the interaction and coordination of activities between different manufacturing sites or different units within the same manufacturing complex (Shobrys and White 2002). Thus, the integration of P + S under an economic objective function is expected to result in cheaper supply chains, with production targets that can be satisfied at the plant site scheduling level.

**Figure 2:**
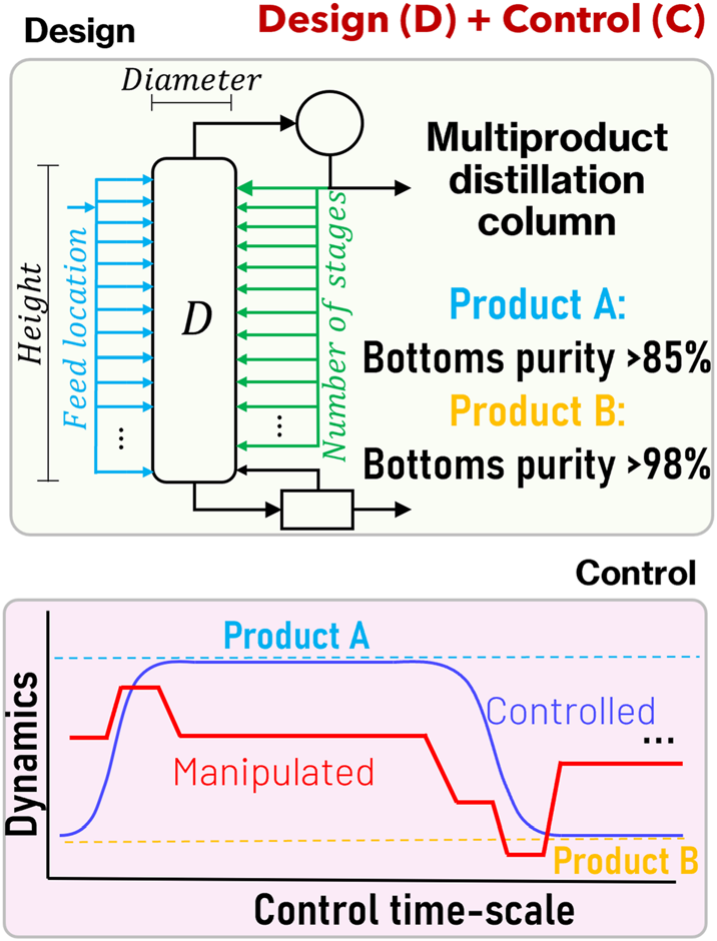
Illustrative design and control problem.

Figure 2 illustrates a D + C problem relevant to MINLP optimization, which is the optimal sizing of a multiproduct continuous distillation column, which in this case has a bottoms product with different purities A and B, e.g., see (Alcántara-Avila et al. 2016; Buitimea-Cerón et al. 2023; Medina-Herrera et al. 2020; Thotla et al. 2007). Both nonlinearities and discrete decisions appear in the design part of this problem due to the consideration of nonlinear mass/energy balances and the presence of discrete variables such as the number of trays and feed location, thus making the design problem by itself an MINLP. Moreover, the consideration of set-point change A→B and B→A or other disturbance scenarios through discretized differential equations make the NLP part of this problem more complex. The solution of this problem with an economic objective function through simultaneous D + C would return a profitable design that is able to accommodate the production of both A and B, while keeping dynamic transitions A→B and B→A feasible and inexpensive. The reader is referred to previous reviews in the field (Burnak et al. 2019a; Pistikopoulos et al. 2024; Swartz and Kawajiri 2019; Yuan et al. 2012; Zhou et al. 2018), which provide further D + C integration examples.

**Figure 3:**
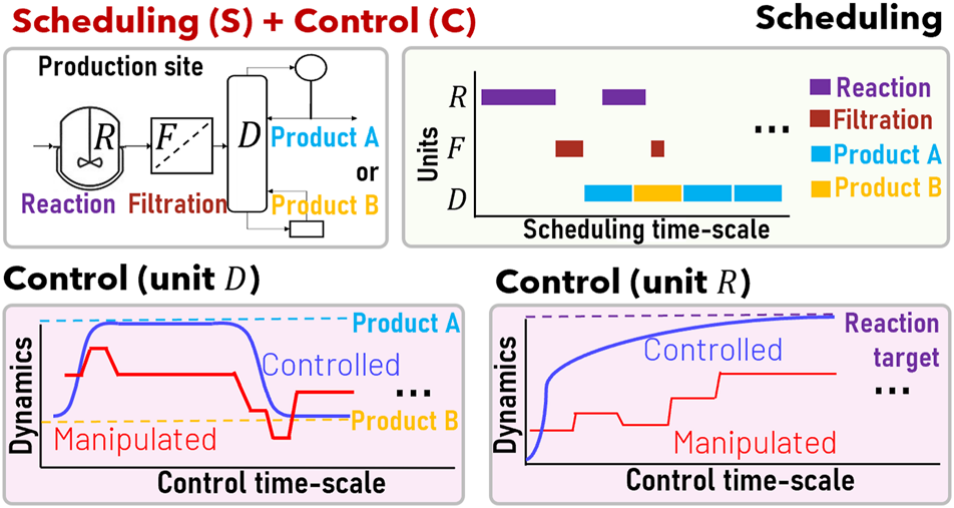
Illustrative scheduling and control problem.

Figure 3 illustrates an S + C problem where a continuous multiproduct distillation column is preceded by two batch operations: reaction and filtration. The scheduling part of this problem introduces discrete decisions when modeling the batching, sequencing, and timing of reaction and filtration tasks, while finding the optimal production sequence of products A and B based on a demand scenario. Furthermore, the consideration of dynamic models to optimize the control actions of key unit operations such as the reactor and the distillation column introduces nonlinear constraints to the formulation, resulting in an MINLP formulation, e.g., see (Chu and You 2013; Nie et al. 2015). The simultaneous S + C of this problem is expected to yield a production schedule that is more likely to satisfy the demand requirements for products A and B, by avoiding the potential inconsistences that may arise with traditional sequential S and C solutions, e.g., not being able to satisfy a reaction production target, due to a selection of batch sizes and processing times in the scheduling layer that is blind to reactor control considerations. The reader is referred to previous reviews in the field (Andrés-Martínez and Ricardez-Sandoval 2022b; Burnak et al. 2019a; Dias and Ierapetritou 2016; Engell and Harjunkoski 2012; Pistikopoulos et al. 2024), which provide further S + C integration examples.

**Figure 4:**
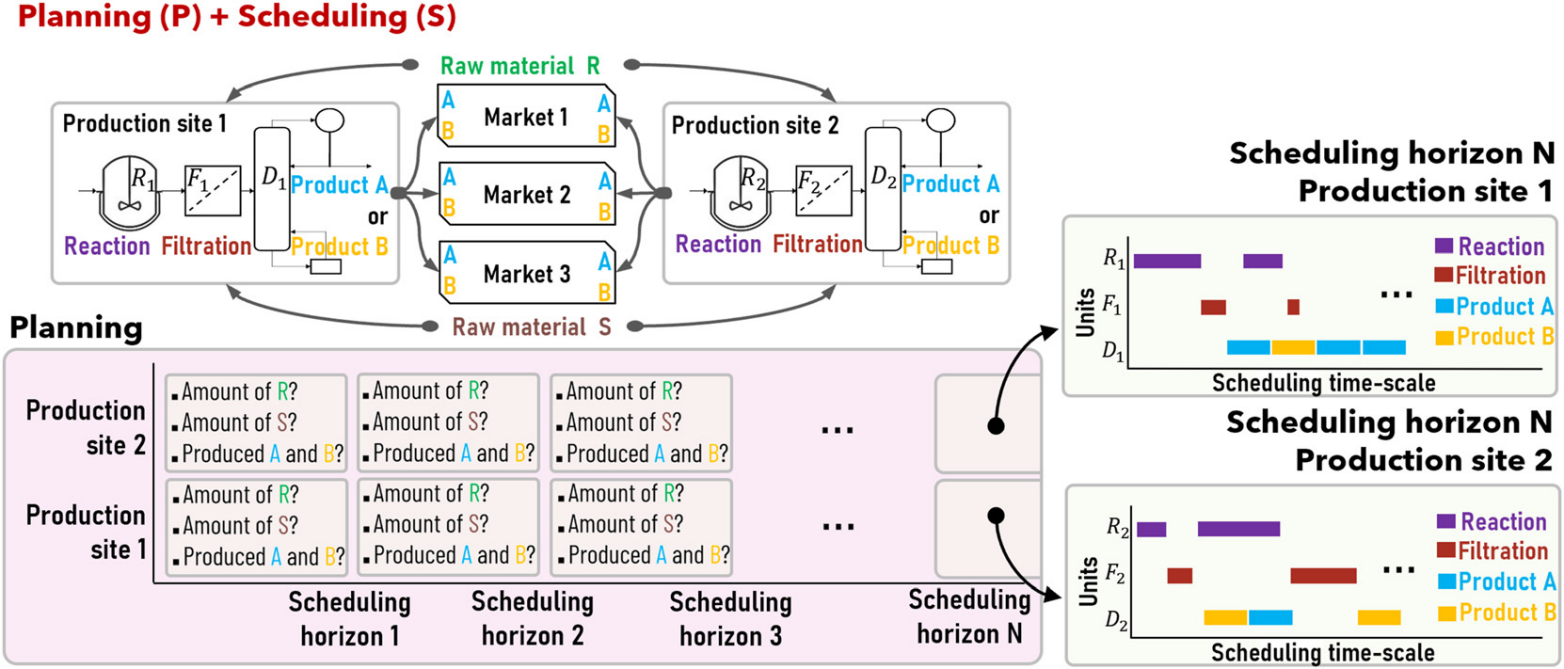
Illustrative planning and scheduling problem.

An example of a P + S problem is shown in Figure 4. This example considers the same multiproduct plant shown in Figure 3, except that it now assumes that there are two production sites available (sites 1 and 2). The planning part of the problem aims to find the optimal amount of raw materials *R* and *S* that must be purchased, as well as the optimal distribution of products within three available markets, based on product demand and raw material availability forecasts. The simultaneous P + S solution thus generates a production plan for the purchase of raw materials and distribution of products within a planning scheduling horizon, as well as the optimal scheduling of tasks at each production site, e.g., see (Birewar and Grossmann 1990; Brunaud et al. 2019). Without P and S integration, planning-defined targets may be infeasible to meet at the scheduling level due to scheduling constraints such as updated demand orders or back-logs that are not considered at the planning level in real time (Shobrys and White 2002). It is noteworthy that most P + S problems result in mixed integer programming (MIP) formulations; however, both the planning and the scheduling layers may introduce nonlinearities, e.g., blend-scheduling (Ahmednooh et al. 2023), crude distillation refinery planning (Grossmann 2012). The reader is referred to previous reviews in the field (Andrés-Martínez and Ricardez-Sandoval 2022b; Phanden et al. 2011; Verderame et al. 2010), which provide further P + S integration examples.

Combinations of the examples discussed above can be used to illustrate the integration of three or more decision layers. For example, combining examples in Figures 3 and 4 results in a problem that simultaneously recognizes P + S + C interactions. For instance, changes in process dynamics at the control timescale in the distillation or the reactor have an impact on task operating times, thus affecting scheduling decisions (e.g., selection of reaction tasks with short processing times that fit within a tight production schedule) and the overall production plan (e.g., readjustments of production capacity in one of the production sites to satisfy scheduling time requirements). Another example of a three-layers integration results when combining D + S + C interactions, where scheduling and control considerations may affect the design of a production plant (e.g., processing units with larger capacities may be needed to operate under pessimistic input material availability and disturbance scenarios). The reader is referred to previous reviews in the field (Andrés-Martínez and Ricardez-Sandoval 2022b; Burnak et al. 2019a; Castro et al. 2018; Nwasuka and Nwaiwu 2024; Pistikopoulos et al. 2024), which provide further P + S + C and D + S + C integration examples.

## 3 General-purpose MINLP solvers

According to Liberti et al. (2021), the motivation to develop general-purpose deterministic MINLP solution algorithms in chemical engineering originated from seminal studies such as those by Geoffrion (1972), who proposed the Generalized Benders Decomposition (GBD) algorithm, and Grossmann (Duran and Grossmann 1986; Grossmann 1985), who were among the first that outlined the handling of nonlinearities in the optimal synthesis of process flowsheets through Branch and Bound (BB) and Outer Approximation (OA) techniques. The reader is referred to the review by Kronqvist et al. (2019) for further details about the working principle of these strategies. Nowadays, there is a variety of commercial and open source general-purpose MINLP solvers (Boukouvala et al. 2016; Bynum et al. 2021; Kronqvist et al. 2019).

Early attempts to address integrated MINLP decision-making problems appeared in the context of optimal design and scheduling of multipurpose batch plants that consisted of finding the optimal number of processing units and their capacities to minimize the total capital cost of the plant, while simultaneously accommodating the production of multiple products within a fixed time horizon (Faqir and Karimi 1989; Vaselenak et al. 1987). These problems were addressed by relying on custom formulations that allowed solving the problems through standard NLP techniques. Later, with the advancement of MINLP algorithms, studies started to emerge in the fields of design and control, planning and scheduling, scheduling and control, and their combinations. Some of these applications are summarized below.

*Generalized Benders Decomposition*: GBD has been used for the simultaneous design and control of separation units including conventional (Bansal et al. 2003; Luyben and Floudas 1994; Mohideen et al. 1996) and heat-integrated (Luyben and Floudas 1994) separation systems. GBD has also been used to solve the optimal design and scheduling of multipurpose batch plants (X. Lin and Floudas 2001). More recently, GBD has been used to address the simultaneous scheduling and control of multipurpose batch plants with network structures (Chu and You 2013; Nie et al. 2015), and the design and control of a reactive distillation column (Vasudevan et al. 2018).

*Branch and Bound:* BB-based strategies have been mostly used to address problems in the field of simultaneous scheduling and control, with applications in multiproduct continuous and batch manufacturing processes (Flores-Tlacuahuac and Grossmann 2010b; Nie et al. 2012), single-unit multiproduct reactors (Zhuge and Ierapetritou 2012), and heat exchanger networks under fouling (Lozano Santamaria and Macchietto 2018). BB has also been used in other fields, e.g., to support the simultaneous design and control of reactor networks (Zhao and Marquardt 2016), or the planning and scheduling of supply chains in the oil and chemical industries (Lasschuit and Thijssen 2004).

*Outer Approximation:* OA techniques have been applied to a broader range of applications, thanks to availability of OA-based solvers such as DICOPT in GAMS, AOA in AIMMS, OAERAP in gPROMS, among others. These applications include the design and control of conventional distillation columns (Bansal et al. 2003), semi-batch polymerization reactors (Asteasuain et al. 2004), a system of interconnected continuous stirred tank reactors (CSTR) (Flores-Tlacuahuac and Biegler 2007), and a semi-continuous distillation columns (Meidanshahi and Adams 2016). For scheduling and control, OA applications include the cyclic scheduling and control of a multiproduct CSTR (Flores-Tlacuahuac and Grossmann 2006) and a polymerization reactor (Terrazas-Moreno et al. 2007). OA has also been applied to multiple design-planning and design-scheduling problems such as the optimal strategic planning and design of a network of petrochemical production processes (Al-Qahtani et al. 2008); the design and scheduling of desalinization systems (Lu et al. 2006; Tanvir and Mujtaba 2008), among others. Furthermore, OA has allowed the integration of three or more decision layers, e.g., the simultaneous planning, scheduling and control of single-unit systems (Gutiérrez-Limón et al. 2014; Y. Peng and Ricardez-Sandoval 2021), the simultaneous design, scheduling and control of a polymerization reactor (Terrazas-Moreno et al. 2008b), and the simultaneous design, planning, scheduling, and control of multiproduct batch plants (Corsano et al. 2009).

Based on the above, the solvers that have been substantially applied in the context of integrated decision-making include DICOPT, which is based on the OA concept; SBB, which is based on the BB principle; and BARON, which is based on BB, constraint propagation and duality techniques that make it suitable for global optimization. Note that GBD (as a general-purpose solver) is closely related and usually requires more iterations than OA; hence, GBD is not widely used in practice for general MINLP optimization. Nonetheless, GBD has been successfully used to exploit special problem structures and tackle integrated decision-making problems (Chu and You 2013; Ji and Gu 2021; Nie et al. 2015). Customized GBD methods leverage the special structure of integrated decision-making problems by separating them into a master problem that considers one of the decision layers (e.g., scheduling) and a subproblem that considers the remainder of the problem (e.g., control decisions). The resulting decomposed problem can then be solved iteratively, i.e., at each iteration the variables of the master problem are fixed in the subproblem, whose solution is used to update the master problem with a new constraint called a Benders cut.

Other general-purpose MINLP solvers include those based on extended cutting planes (Westerlund and Pettersson 1995) and extended supporting hyperplanes (Kronqvist et al. 2016); however, these have not been widely applied in the context of integrated decision-making. Similarly, Generalized Disjunctive Programming (GDP) modeling and solution strategies were proposed to address some limitation of MINLP techniques, i.e., dependence on a predefined algebraic formulation such as big-M (Chen et al. 2022), and numerical issues encountered due to the interactions of continuous and discrete decisions (Chen et al. 2021; Ruiz and Grossmann 2017). GDP provides a logical modeling framework, where switching decisions between groups of constraints are represented through Boolean variables that activate/deactivate constraints within disjunction terms (Chen et al. 2022). GDP applications in integrated decision-making are also scarce; nevertheless, a few studies have been published in the fields of optimal process design and optimal process scheduling, e.g., see (Mencarelli et al. 2020; Raman and Grossmann 1994). A GDP formulation is often preferred over a standard MINLP formulation because of its generality, i.e., this formulation offers flexibility to transform the problem into an MINLP through a variety of reformulations, with Big-M and convex hull being the most widely used in the literature (Grossmann 2002; Grossmann and Lee 2003). These advances have led to the development of Pyosyn: A framework for conceptual design modeling and optimization that facilitates superstructure optimization by relying on the Pyomo.GDP environment (Chen et al. 2021). Another tool based on the GDP paradigm was proposed to generate hybrid first-principles and surrogate models for superstructure optimization (Pedrozo et al. 2021). An example of an integrated decision-making GDP application is the design, operation, and expansion planning of chemical processes (van den Heever and E. Grossmann 1999). The results obtained from this application show significant reductions in the number of MIP iterations needed by a logic-based OA and a tailored logic-based decomposition algorithm, where “logic-based” is used to indicate that these algorithms exploit the disjunctive structure of the formulation without the need of an MINLP reformulation, i.e., see Section 5.5.

Despite the success of general-purpose MINLP solvers, several studies have demonstrated the drop in performance or even failure of general-purpose MINLP solvers and highlighted the need of more efficient solution strategies when dealing with integrated decision-making problems, e.g., see (Akulker and Aydin 2023; Cafaro and Grossmann 2014; Chu and You 2013; Liu et al. 2012; Ma et al. 2021). To mention a few examples, Flores-Tlacuahuac and Biegler (Flores-Tlacuahuac and Biegler 2007) compared the performance of SBB and DICOPT when tackling the simultaneous design and control of two CSTRs in series with up to 2,647 continuous variables and 3,905 constraints. That study showed that DICOPT led to solver failure in some instances, due to poor linearization of nonconvex functions. Valdez-Navarro and Ricardez-Sandoval (Valdez-Navarro and Ricardez-Sandoval 2019) compared the performance of SBB, DICOPT, and BARON to address the simultaneous scheduling and control of network batch processes under uncertainty and found that increasing the number of uncertain scenarios has a negative impact on traditional solver performance. The most challenging case study they tested involved 361 realizations in 2 uncertain parameters, 446,271 continuous variables, 66 binary variables, and 450,365 constraints. The authors found that SBB, DICOPT, and BARON failed to identify a feasible solution to the problem after 1 day of computation, even though the problem was known to be feasible. Lozano-Santamaria and Macchietto (Lozano Santamaria and Macchietto 2018) found that standard general-purpose MINLP solvers may be time intensive and require solution times in the order of magnitude of days when solving realistic simultaneous scheduling and control problems of heat exchanger networks under fouling; thus, more efficient strategies were suggested to find solutions in shorter computational times.

Generally speaking, traditional MINLP solvers may require lengthy, and in some cases prohibited, computational times, they may only identify low-quality local solutions (e.g., get stuck at the initialization point), or even fail to find feasible solutions, e.g., when dealing with the absence of phases and linearizing at zero flows in problems involving optimal design (Ghouse et al. 2018). Typically, one of the key factors affecting general-purpose solvers’ performance is the convexity/nonconvexity characteristics of the problem at hand and how these characteristics may lead to different local solutions depending on the initialization. A second factor is the type and number of nonlinear expressions that are part of the formulation, and how these may impact the solver’s success to detect feasible solutions, or run into numerical instabilities. A third factor is the number of discrete variables in the problem and their interactions with continuous variables, leading to large problems with challenging combinatorial structures. In the authors’ experience, the growth in problem size when different layers are integrated is highly dependent on the specific application and the features included in the formulation. For example, the optimal design of a catalytic distillation column at steady state using the rigorous material, equilibrium, summation, and enthalpy (MESH) equations for a superstructure with 22 potential stages and four components resulted in 2,585 continuous variables, 305 inequality constraints, and 2,584 equality constraints (Liñán et al. 2020). When including four dynamic transitions between different product qualities, the number of continuous variables and constraints of this problem increased by two orders of magnitude (Liñán and Ricardez-Sandoval 2022b). In addition to the effect of coupling decision layers, the problem size can also be affected when increasing the number of uncertainty parameters and realizations, e.g., in a simultaneous scheduling and control problem of a network batch plant, the number of constraints increased from 3,029 to 450,365 when increasing the number of uncertainty realizations from 1 to 361 (Valdez-Navarro and Ricardez-Sandoval 2019). Another factor that affects the size of integrated decision-making problems is the user flexibility in the selection of time horizons. For instance, the size of an integrated production planning and scheduling problem for a multisite-multiproduct process increases as the number of planned time periods increases, e.g., a problem with five time periods has 720 binary variables, 4,006 continuous variables, and 10,465 constraints, while a problem with 90 time periods has 12,960 binary variables, 72,091 continuous variables, and 188,455 constraints (Shah and Ierapetritou 2012). The aforementioned issues have motivated the development of alternative deterministic MINLP solution techniques for integrated decision-making, this is discussed next.

## 4 Specialized MINLP techniques for integrated decision-making

**Figure 5:**
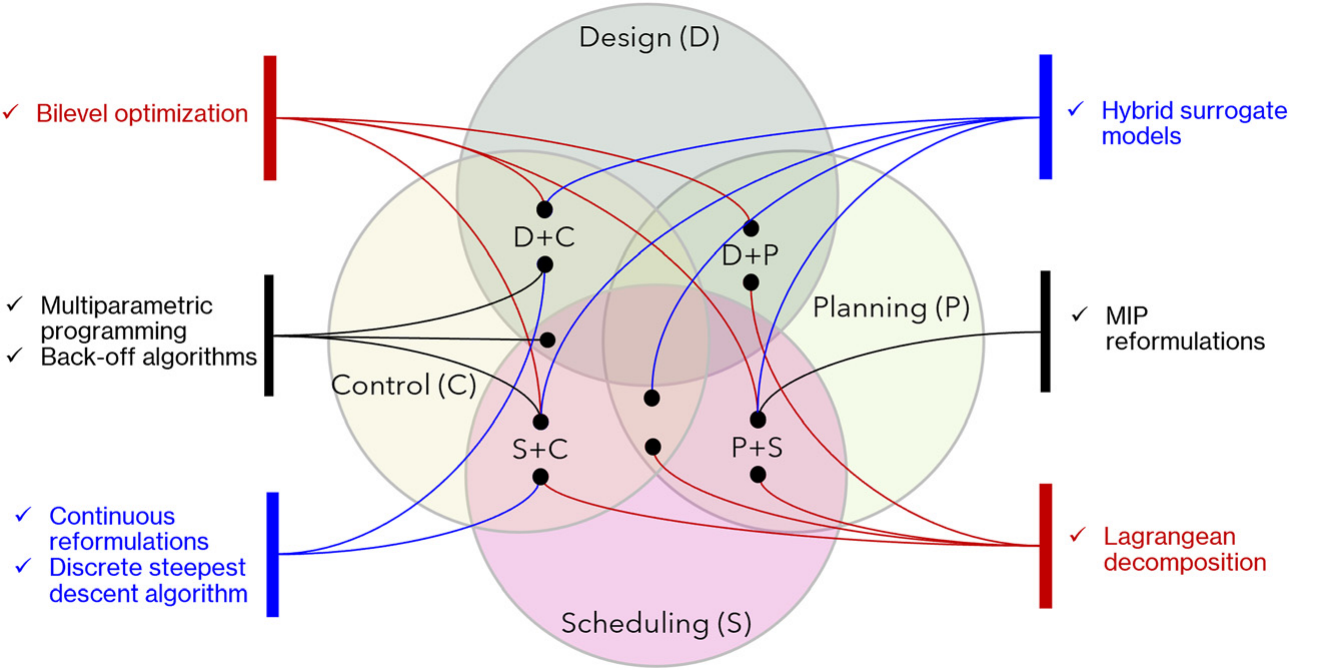
Algorithms and tools for integrated decision-making. Note that this diagram only shows existing applications for problems resulting in MINLP formulations.

This section provides an overview of the MINLP algorithms and frameworks for integrated decision-making (i.e., other than those mentioned in the previous section) including mixed integer programming (MIP) reformulations, hybrid surrogate models, continuous reformulations, Lagrangian relaxation, and the discrete steepest descent algorithm. Other relevant tools included in this section are multiparametric programming, bilevel optimization, and back-off algorithms. These three strategies do not necessarily focus on facilitating the solution of an underlying single-level MINLP problem resulting from the integration; instead, these techniques aim to facilitate the incorporation of other features that arise in integrated decision-making, such as the presence of conflicting objectives, uncertainties, and disturbances. Figure 5 summarizes the optimization tools discussed throughout this section, and their main MINLP integrated decision-making applications.

### 4.1 MIP reformulations

In MIP reformulations, nonlinearities are reformulated as linear relationships between integer and continuous variables. MIP reformulations of MINLP problems may involve exact linearization, e.g., when linearly reformulating the multiplication of two binary variables, or the multiplication of binary and continuous variables. Similarly, another example emerges when continuous variables in nonlinear models are restricted to take discrete values thus resulting in an MIP formulation (Voudouris and Grossmann 1992). Exact linearization have been successfully applied to integrated scheduling and planning problems involving batch processing plants (Orçun et al. 2001; Tan and Khoshnevis 2004). In more general scenarios, exact linearization transformations may not be available and MIP input–output approaches that only provide an approximation of the original MINLP may be needed. These approaches rely on the approximation of nonlinearities as linear operators, leading to a formulation that can be readily solved with MIP methods, at the expense of having a model that is valid within a reduced space where the linearization was performed. This approach has been successfully applied to capital investment planning and scheduling of an oil refinery (Menezes et al. 2015).

### 4.2 Hybrid surrogate models

Hybrid surrogate models combining data-driven and first-principles models have also been considered to approximate one or some of the decision layers of the original integrated MINLP problem, e.g., using a data-driven surrogate model to approximate process dynamics in simultaneous design and control (Ricardez-Sandoval et al. 2009; Sachio et al. 2021; Trainor et al. 2013). Surrogate models aim to provide an accurate approximate model of some decision layers using sampled input–output data taken over a search region of interest, i.e., they aim to be valid over the whole optimization region of interest. The surrogate model itself can take the form of a linear model, a piecewise linear model, a polynomial model, a neural network, among others; consequently, the resulting hybrid surrogate model may remain as an MINLP that is often solved using general-purpose solvers. For instance, the integration of design and control through the explicit representation of the optimal controller using reinforcement learning has been proposed in the literature (Sachio et al. 2022). The approximation of process dynamics has also been considered for the integration of scheduling and robust model predictive control, through the use of two surrogate models in the form of artificial neural networks: one for the identification of the feasible space of operation, and the other for the approximation of the state and input variables of the dynamic system (Dias and Ierapetritou 2020).

Many hybrid surrogate models can also be regarded as MIP reformulations. For example, piecewise linear reformulations of data-driven low-order models (e.g., Hammerstein–Wiener models, and piecewise affine models) were proposed to represent nonlinear process dynamics in simultaneous scheduling and control problems, resulting in an MIP integrated problem (Simkoff and Baldea 2019). This strategy has been tested for instance on a continuous CSTR with cyclic production (Zhuge and Ierapetritou 2015), a continuous multiproduct polymerization reactor (Kelley et al. 2018a), and an air separation unit (Kelley et al. 2018b). A similar strategy was employed to simultaneously consider planning, scheduling, and dynamic optimization of batch processes, where dynamic models were replaced by linear surrogate models, resulting in an MIP formulation (Chu and You 2014a). Piecewise linear approximations that transform an MINLP into an MIP have also been considered for the planning and scheduling of flexible process networks under uncertainty (Yue and You 2013). Overall, hybrid surrogate methods may simplify the effort of model development and speed-up the solution of the optimization problem; however, they require training and validation of data sets that are assumed to cover/predict the entire operating range of the plant/model.

### 4.3 Continuous reformulations

In continuous reformulations, discrete decisions are expressed as continuous variables, and discrete requirements are enforced via additional constraints. Continuous reformulations have been developed to reformulate MINLPs as Nonlinear Programs (NLPs) to reduce the high computational costs often faced by MINLP and MIP techniques. Examples of reformulations include complementarity constraints, circle constraints, nonlinear complementarity problem functions, among others (Baumrucker et al. 2008; Burre et al. 2023; Stein et al. 2004). These techniques often require the iterative optimization of the resulting NLP with varying tolerance parameters that either enforce integer requirements as they go to zero or the consideration of integer requirements through objective function penalization. Despite this, they are usually faster than traditional MINLP solvers. Integrated decision-making MINLP applications include the integration of optimal scheduling and control of heat exchanger networks under fouling (Santamaria and Macchietto 2019), scheduling and NMPC of a multiproduct continuous reactor (Andrés-Martínez and Ricardez-Sandoval 2022a), simultaneous design and control of plug flow and CSTR reactor networks (Zhao and Marquardt 2017), design and control of extractive distillation column systems (Ramos et al. 2014).

When using NLP local solvers, the main drawback of continuous reformulations is that they inevitably lead to a nonconvex optimization problem that generally requires educated initial guesses to achieve convergence (Stein et al. 2004). Alternatively, when a global solver such as BARON or MAiNGO is used, case studies involving superstructure design have demonstrated that, from a computational performance point of view, an MINLP superstructure formulation is usually desirable over its continuous reformulation for problems with convex functions. However, when solving nonconvex MINLPs with the same global solvers, continuous reformulations may result in smaller problems and tighter relaxations (Burre et al. 2023). Despite these preliminary advantages, the performance of continuous reformulations using global solvers for integrated decision-making problems has not been reported in the literature, presumably due to the long computational times that may be required when compared to local NLP solvers.

### 4.4 Lagrangian relaxation

Lagrangian relaxation corresponds to the general idea of penalizing a subset of complicating constraints in the objective function of an optimization problem, i.e., complicating constraints are dualized. The nonnegative weights used to penalize these constraints are known as Lagrangian multipliers (Guignard 2003). An application of Lagrangian relaxation that has become relevant in the context of MINLP integrated decision-making in chemical engineering is known as Lagrangian decomposition (LD), which is a useful approach to exploit optimization problem structures where two (or more) subset of constraints interconnected through common variables appear in the problem formulation. For instance, in simultaneous scheduling and control, some constraints and variables are used to model scheduling decisions, and others are used to capture transient operation, i.e., dynamic path constraints. These two groups of constraints are often connected through common variables. In the case of two subsets of constraints, if common variables are duplicated, the linking constraints that connect these variables can be dualized resulting in a decomposable formulation where each subset of constraints considers independent variables (Guignard 2003).

Note that the value of Lagrangian multipliers used to penalize constraints is initially unknown. Hence, once the problem is decomposed, an adaptive approach is used to update the Lagrangian multipliers. This adaptive approach is incorporated within the main iterative process of LD, which is as follows: 1) the solution of the two independent problems is used to compute a lower bound to the problem and 2) a problem with fixed integer variables that returns an upper bound to the problem is computed. For example, chemical engineering applications in the field of planning, scheduling, and control of a multiproduct CSTR (Mora-Mariano et al. 2020), or the scheduling and control of polymerization reactors (Terrazas-Moreno et al. 2008a) have implemented the subgradient method developed by Fisher (1985) to iteratively find and update Lagrangian multipliers. Other relevant works in the field include the simultaneous supply chain design and planning in the electric motor industry (Yongheng et al. 2014), and the integration of refinery planning and crude-oil scheduling (Mouret et al. 2011; Yang et al. 2020).

In general, LD helps to reduce the computational cost of solving integrated decision-making problems, against the direct solution without decomposition techniques (Andrés-Martínez and Ricardez-Sandoval 2022b). Note that there are some challenges in the application of LD to MINLP integrated decision-making. For instance, an adequate strategy should be used to update Lagrangian multipliers, e.g., the algorithm may not converge if the subgradient step size is not defined or updated properly. Furthermore, problem-specific heuristics may be needed to solve the resulting MINLP subproblems efficiently (Mouret et al. 2011). In addition, and due to the presence of discrete variables and nonconvexities, the duality gap obtained at each iteration is only expected to become small enough to obtain near-optimal heuristic solutions, but it is not guaranteed to reach a value near zero, i.e., the lower bound may remain strictly lower than the true optimum of the problem (Mora-Mariano et al. 2020; Mouret et al. 2011).

### 4.5 Discrete steepest descent algorithm

Concepts from discrete convex analysis (Murota 2003) can be incorporated into novel decomposition-based algorithms particularly tailored to handle ordered discrete decisions, i.e., integer variables and ordered discrete structures of Boolean or binary variables. Variables of this type appear frequently in integrated decision-making MINLP problems, e.g., number of units in series/parallel in a reactor superstructure; selection of the most sensitive stage for distillation temperature control applications; number of times a task is executed during a production schedule. The key characteristic of algorithms based on discrete convex analysis is that they verify local optimality of ordered discrete decisions by performing objective function evaluations over a discrete neighborhood of a solution candidate, while the optimality of the remaining variables of the problem is determined by a general-purpose solver. As a result, these algorithms may yield locally optimal solutions that may not be found with traditional MINLP methods. An algorithm that follows these principles is the discrete steepest descent algorithm (D-SDA) (Liñán and Ricardez-Sandoval 2024a).

In the context of MINLP integrated decision making, the D-SDA has been applied for the simultaneous design and control of conventional and catalytic distillation columns (Liñán and Ricardez-Sandoval 2022a; Palma-Flores et al. 2023), and the simultaneous scheduling and dynamic optimization of network batch processes (Liñán and Ricardez-Sandoval 2024a). For these applications, D-SDA converged to local optima with objective function values that are better than those found with general-purpose MINLP solvers in shorter computational times. Note that the local optimality guarantees of D-SDA rely on the exploration of a neighborhood (i.e., an integrally convex neighborhood) whose size increases exponentially with the number of ordered discrete decisions. Integrally convex neighborhood explorations may lead to lengthy computational times; thus, using subsets of the integrally convex neighborhood helps to speed up convergence but may worsen the objective function value attained.

### 4.6 Multiparametric programming

Multiparametric programming (MP) is a tool used to systematically analyze the effect of varying parameters (e.g., uncertainties, disturbances, design parameters) in optimization problems (Pistikopoulos et al. 2007). Multiparametric optimization has played a significant role in the solution of optimization problems arising in plantwide integrated decision-making. This technique aims to find exact offline solutions to (or part of) the MIDO problems arising in integrated decision-making, by deriving explicit maps of optimal decisions, i.e., the parameter space is partitioned into subregions known as “critical regions,” whereas optimization variables are expressed as a piecewise function of the parameters. Since optimal decision maps are derived offline, these can be readily incorporated within online feedback decision loops, e.g., Explicit Model Predictive Control (MPC). Thus, one of the main features offered by multiparametric optimization is the possibility of replacing parts or the complete optimization problem by piecewise function evaluations (Pistikopoulos 2012).

Relevant MP applications include integrated design, scheduling ,and control, with applications in the operation of a multiproduct continuous and batch reactors (Burnak et al. 2019b; Burnak and Pistikopoulos 2020; Zhuge and Ierapetritou 2014), and the design and control of conventional and reactive distillation systems (Sakizlis et al. 2003; Tian et al. 2021). Different decomposition methodologies have been proposed depending on the application, e.g., embedding closed-loop MPC dynamics in the scheduling formulation via MP; deriving offline multiparametric expressions for both scheduling and MPC control interconnected through bridging models; considering design variables as parameters in a multiparametric MPC problem to enable design and control integration, among others (Pappas et al. 2021). Despite their successful applications, one of the main challenges in MP is to overcome its dimensionality limitations. For instance, determining critical regions generally involves the solution of multiple optimization subproblems with fixed parameters, which may be computationally intensive for general MIDO systems involving multiple states, e.g., increasing the number of decision variables in a multiparametric MPC formulation leads to an exponential increase in the number of critical regions (Burnak and Pistikopoulos 2020; Pappas et al. 2021).

### 4.7 Bilevel optimization

Bilevel optimization plays a key role in integrated decision-making problems where two decision layers consider different objectives. In a bilevel optimization formulation, a lower-level optimization problem is embedded within an upper-level optimization problem. One example is a lower-level model predictive controller (MPC) that aims to optimize set-point tracking and an upper-level scheduling layer that aims to minimize production costs. In contrast to sequential solution techniques where different decision layers are optimized independently, bilevel methods aim to enable the interaction between the decision layers involved. Chemical engineering applications include the simultaneous scheduling and dynamic optimization of batch plants and dynamic transitions in a polymer manufacturing process, where dynamic optimization problems are solved *a priori* to obtain a piecewise response function of the lower-level problem, which is latter embedded into the upper-level and solved as single-level optimization problems (Chu and You 2012, 2014b). The reformulation of a bilevel problem into a single level has also been proposed in the context of integration of planning and scheduling under demand uncertainty, through surrogate-based and data-driven techniques (Beykal et al. 2022). A bilevel decomposition approach has also been proposed for the simultaneous planning and scheduling of single-stage multiproduct continuous plants with parallel lines, with an upper-level planning and relaxed scheduling model and a lower-level simultaneous planning and scheduling model (Erdirik-Dogan and Grossmann 2008).

Another approach recently proposed in the literature integrates NMPC with process design, through the Karush–Kuhn–Tucker (KKT) transformation of the NMPC optimization layer. This leads into a highly nonconvex single-level problem that has the potential to be solved with conventional optimization solvers. In the context of MINLP, this strategy has been used to address the simultaneous design and control of distillation units (Palma-Flores et al. 2023). Note that the solution of a bilevel optimization problem reformulated as a single level problem brings additional challenges, e.g., the single-level formulation that results from using the KKT transformation violates multiple constraint qualifications at every feasible point, due to the KKT complementarity slackness constraints. Moreover, an optimal solution for the reformulated single-level problem may not represent an optimal solution for the original bilevel problem (Dempe et al. 2015).

Note that customized decomposition algorithms for single-level MINLP optimization are also referred in the literature as bilevel optimization approaches, e.g., Lara et al. (2019) deal with the MINLP design and planning of manufacturing networks through a customized decomposition that result in an MIP master problem and NLP subproblems.

### 4.8 Back-off algorithms

Back-off algorithms have been used to effectively handle uncertainties and disturbances in optimal process integration. When uncertainty is not considered in optimal decision-making, the solution typically lies in the boundary of the feasible space of operation (Patilas and Kookos 2021b). Consequently, uncertainty and slight disturbance variations may shift the operation of a process outside their feasible operating window, e.g., weeping may occur in a distillation column. Back-off algorithms iteratively move away from an initial nominal solution to a new optimal and feasible operating point in the presence of uncertainty/disturbances. A back-off algorithm serves as a more suitable technique to deal with MINLP optimization problems under uncertainty, when compared to traditional uncertainty handling strategies in optimization, e.g., the direct solution of the monolithic multiscenario MINLP formulation using general-purpose solvers (C. Li and Grossmann 2021).

The main challenge in this approach is to determine a systematic strategy to determine the amount of back-off needed to accommodate uncertainties and disturbances in the operation. In the context of MINLP integrated decision-making, the back-off approach has been applied to the simultaneous design and control of a CSTR, an ideal distillation column, and a reactive distillation process (Patilas and Kookos 2021b, 2021a). The amount of back-off was determined iteratively by finding the maximum violation of the vector of inequality constraints for a given time horizon and disturbance scenario, i.e., a worst-case approximation. Aiming to avoid conservative solutions due to the worst-case approximation, a stochastic back-off approach that models disturbance and uncertainty with stochastic random variables has been proposed and applied to the simultaneous scheduling and dynamic optimization of batch plants (Valdez-Navarro and Ricardez-Sandoval 2019), and the design, control, and scheduling of a multiproduct reactor (Koller et al. 2018). Note that further research is still needed to overcome the stochastic back-off computational challenges, e.g., due to its random nature, the stochastic back-off approach may yield different solutions every time the algorithm runs, and the need to run Monte Carlo simulations for back-off quantification remains the key computational bottleneck of this approach.

## 5 Recent MINLP algorithmic developments and perspectives

As discussed above, significant progress has been made in the development of MINLP optimization tools for integrated decision-making. Despite these efforts, the existing techniques still exhibit limitations, e.g., convergence issues in continuous reformulations and Lagrangian decomposition, or accuracy issues with simple linearization-based MIP reformulations. Thus, despite the advances in the field, more research is still needed in the development of MINLP algorithms that can efficiently handle integrated decision-making problems. For instance, more than 80 % of the works discussed in the previous section (Figure 5) link two decision layers; however, the integration of three or more layers is still limited due to problem sizes and complexity, e.g., while simultaneous scheduling and control problems often deal with chemical plants involving multiple processing units, simultaneous planning scheduling and control problems typically focus on a single processing unit, e.g., see the review by Andrés-Martínez and Ricardez-Sandoval (2022b).

**Figure 6:**
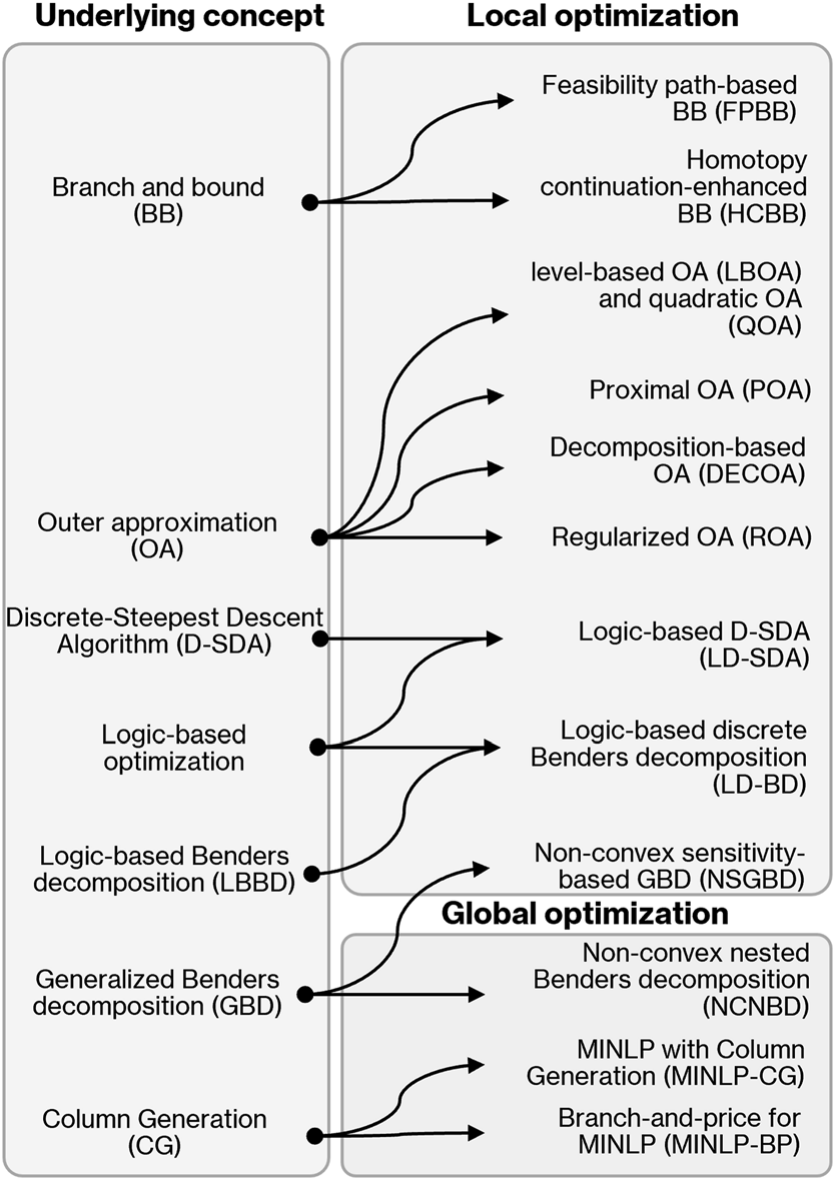
Recently proposed algorithms in the field of deterministic MINLP optimization.

This section reviews recent model-based MINLP algorithms that have not been explored in the context of optimal process integration and that may be attractive to solve problems in this field given their modeling features. Accordingly, this section provides insights on how these MINLP techniques can be potentially explored to propose new and more efficient algorithms for optimal integrated decision-making. Note that there have been a significant number of studies in this field in recent years; hence, we only focus on MINLP local and global algorithms proposed within the last 5 years. Although previous MINLP reviews do not focus on optimal process integration (Boukouvala et al. 2016; Burer and Letchford 2012; D’Ambrosio and Lodi 2013; Kronqvist et al. 2019; M.-H. Lin et al. 2012), they discuss studies from previous years and decades not covered in this section. The algorithms discussed throughout this section are summarized in Figure 6, which classifies them according to their local or global MINLP optimization characteristics and their underlying principle. The benefits that these algorithms could bring to the field of optimal process integration and some potential applications discussed through this section are summarized in Table 1. As shown in this table, a few of the open research challenges of these algorithms have been outlined. At the end of this section, we provide a short discussion on software tools for integrated decision-making, on the basis of MINLP algorithms.

**Table 1:** Process integration benefits and potential applications of recent MINLP algorithms.

Algorithm	Key features	Potential applications in optimal process integration	Research challenges
Feasibility path-based BB (FPBB) and homotopy continuation-enhanced BB (HCBB)	Facilitate convergence of problems involving nonlinear systems	Intensified distillation-based models for optimal design and control	Handle zero-flow convergence difficulties (Ma and Li 2022)
Level-based OA (LBOA), quadratic OA (QOA), and regularized OA (ROA)	Improve the exploration of discrete variables through regularization	Speed-up convergence in closed-loop scheduling and control schemes	Explore iterative update policies for hyperparameters and the Hessian of the Lagrangian (Bernal et al. 2022b)
Proximal OA (POA)	Aid in the identification of feasible solutions for challenging problems	Problems involving PDEs, e.g., scheduling and control of tubular reactors	Study the effect of using different hyperparameters depending on the problem (De Mauri et al. 2020)
Decomposition-based OA (DECOA)	Exploit block-separable structures in integrated decision-making	Simultaneous planning, scheduling, and dynamic optimization problems	Improve efficiency by adding multiple supporting hyperplanes per iteration (Muts et al. 2020)
Nonconvex sensitivity-based GBD (NSGBD)	Guarantee convergence to a point that satisfies KKT conditions	Existing GBD applications, e.g., scheduling and dynamic optimization of batch processes	Improve performance through parallel computation techniques (J.-J. Lin et al. 2023)
Nonconvex nested Benders decomposition (NCNBD)	Solve multistage nonconvex MINLP problems to global optimality	Simultaneous planning and scheduling of power systems under market price uncertainty	Develop more efficient methods to solve the Lagrangian dual problem (Füllner and Rebennack 2022)
MINLP with column generation (MINLP-CG)	Guarantee convergence to a global optimum for nonconvex problems	Existing LD applications, e.g., simultaneous refinery planning and crude-oil scheduling	Propose performance improvements through the solution of subproblems in parallel (Muts et al. 2023)
Branch-and-price for MINLP (MINLP-BP)	Exploit block-separable structures and provide global optimality convergence guarantees	Simultaneous scheduling and dynamic optimization problems	Speed-up the solution of subproblems when their size increases to remain competitive with respect to existing methods (Allman and Zhang 2021)
Logic-based D-SDA (LD-SDA) and logic-based discrete Benders decomposition (LD-BD)	Incorporate concepts from discrete convex analysis and LBBD into the direct solution of GDP problems	Integrated decision-making problems with GDP formulations and ordered disjunctions	Generalize these approaches to cover a wider variety of GDP formulations (Liñán and Ricardez-Sandoval 2023)

### 5.1 Branch and bound

Based on the BB principle, Ma and Li (2022) proposed the homotopy continuation-enhanced BB (HCBB) algorithm. The key novelty of this strategy is the introduction of a homotopy path between a node and its parent node that improves the convergence and efficiency of solving NLP subproblems through the branching tree. Thus, this feature exploits the potential similarity between the NLP subproblems solved in BB to generate improved variable initializations. As highlighted in that work, this reduces the variability in the local solution obtained for strongly nonconvex MINLPs when testing different initializations, compared to the SBB and DICOPT solvers. Consequently, this feature may benefit the optimization of integrated decision-making MINLP problems, whose NLP subproblems are in general nonconvex and challenging to converge.

Despite the benefits of HCBB, an issue not considered by this algorithm is the convergence to a feasible solution at the root node of the branching tree, i.e., HCBB assumes that good initial guesses are available at the root node. To circumvent this issue, Liu et al. (2022) proposed the feasible path-based BB (FPBB) algorithm, which brings improvements with respect to HCBB by incorporating a feasible path-based framework that partitions continuous variables into independent and dependent variables. By expressing dependent variables as implicit functions of independent variables, the equality constraints linking these variables can be ignored during the BB procedure and convergence is improved. The authors illustrate the benefits of FPBB with different case studies involving separation systems involving multiple interconnected conventional distillation units modeled through nonideal tray-by-tray models. Thus, FPBB is a promising strategy to incorporate highly nonlinear modeling features such those arising in separation systems into integrated decision-making MINLP problems. Accordingly, these algorithmic improvements may be helpful to address integrated decision-making problems involving intensified or heat integrated distillation sequences modeled with rigorous first-principles models, which have been rarely considered in the literature due to their complexity, e.g., see (Dias and Ierapetritou 2019).

### 5.2 Outer approximation

During the last years, several improvements to the standard OA technique have been recently proposed, e.g., level-based OA (LBOA) (Kronqvist et al. 2020), quadratic OA (QOA) (Kronqvist et al. 2020), proximal OA (POA) (De Mauri et al. 2020), decomposition-based OA (DECOA) (Muts et al. 2020), and regularized OA (ROA) (Bernal et al. 2022b). LBOA and QOA aim to accelerate OA by carefully choosing integer combinations in the master problem. In the case of LBOA, it differs from OA by considering two steps when generating trial integer combinations: the solution of an MIP, and the solution of an auxiliary Mixed Integer Quadratic Problem (MIQP) that introduces a regularization based on the Euclidean norm to each iteration to keep the next trial integer combination close to the previous one. QOA follows the same principle as LBOA, but it also incorporates second-order derivative information when choosing trial integer combinations. Test instances demonstrated the computational advantages of LBOA and QOA when solving nonlinear convex MINLPs (Kronqvist et al. 2020). More recently, ROA generalized the regularization idea introduced by LBOA to consider a user-specified regularization function (Bernal et al. 2022b). Due to the success of OA methods, LBOA, QOA, and ROA may benefit integrated decision-making by speeding-up the convergence in the solution. Specifically, the regularization ideas from the algorithms discussed above can be incorporated into the solution of integrated decision-making problems. For instance, subproblem convergence in decomposition methods (e.g., those based on GBD or OA) may be slow when the discrete variables generated by the master problem are not carefully selected as the iterations progress. This poses limitations in the context of integrated decision-making if there is a computational time limit that must be satisfied, e.g., in closed-loop scheduling and control, where decisions must be made in the order of magnitude of the controller sampling time in a bottom-up integration scheme (Caspari et al. 2020). To tackle this issue, regularization techniques may help to keep integer combinations close to an incumbent solution, resulting in subproblems that are similar to each other. This means that subproblems may have access to better initializations and converge faster, if subproblem variables are reinitialized from previous subproblems.

Another recent development is the feasibility pump implemented within OA solvers such as DICOPT, which facilitates the identification of feasible solutions in a preprocessing step (Bernal et al. 2020). POA improves the feasibility pump by integrating the feasibility pump in OA as it iterates. At each iteration, the discrete trial point selection by the OA’s master problem solely relies on improving the objective function of an MIP approximation of the problem; instead, POA balances objective function improvement and feasibility by generating guesses that are closer to the nonlinear feasible space. One disadvantage of POA is the need to tune parameters that define the trade-off between objective function values and constraint feasibility. However, a careful selection of tuning parameters in POA showed superior performance when compared to OA and the execution of the feasibility pump at preprocessing (De Mauri et al. 2020). These improvements are relevant in integrated decision-making problems, where identifying feasible solutions usually requires optimization expertise to develop custom feasibility algorithms depending on the application. For example, the initialization of the single-level MINLP problem in integrated design and NMPC control through bilevel decomposition requires educated initial guesses due to the nonconvexity of complementarity constraints (Palma-Flores et al. 2023). Another example includes the initialization of problems involving partial differential equations (PDE), resulting in mixed-integer PDE-constrained nonlinear optimization formulations that may be difficult to solve without customized initializations, e.g., when considering the scheduling and control of tubular reactors (Flores-Tlacuahuac and Grossmann 2010a), or oilfield production planning and control problems (Aristizabal et al. 2023). Thus, feasibility detection improvements may foster the application of MINLP by engineering practitioners that are not necessarily familiar with feasibility and variable initialization strategies.

The recently proposed DECOA algorithm presents an OA variant that relies on a block-reformulation of the original MINLP problem. This reformulation poses the original problem as multiples blocks of nonlinear constraints and variables interconnected through coupling constraints. Compared to OA, DECOA simplifies the solution of subproblems as multiple smaller subproblems that can be solved in parallel. Compared to OA, DECOA generally reduces the total number of MIP master problems that need to be solved (Muts et al. 2020). Note that integrated decision-making problems may have block-separable structures without the need of additional reformulations; e.g., Kelley et al. (2018a) have identified and exploited block-separable structures for simultaneous scheduling and dynamic optimization, while Li and Ierapetritou (2010) have identified this structure in the integration of planning and scheduling. Thus, DECOA may be a suitable strategy to address decision-making problems integrating multiple (e.g., more than three) decision layers by exploiting their block structure. Note that the OA techniques discussed herein impose the requirement that the MINLP must be convex to guarantee global optimality. In this regard, previous works have attempted to generate cuts using convex and concave McCormick relaxations, solve NLP subproblems to global optimality, and avoid cycling through no-good integer cuts, thus allowing OA to guarantee global optimality (Kesavan et al. 2004; Z. Peng et al. 2024).

### 5.3 Generalized Benders decomposition

Variants of GBD have also been proposed in recent years. Nonconvex sensitivity-based GBD (NSGBD) (J.-J. Lin et al. 2023) is a recent technique that offers convergence improvements compared to GBD. Traditional GBD techniques have been criticized in the past due to its convergence properties, e.g., for a nonconvex NLP problem, GBD may converge to a point that does not satisfy KKT conditions (Geoffrion 1972). NSGBD addresses this issue and guarantees convergence to a KKT point for nonconvex problems. To do that, NSGBD manipulates Benders cuts to guarantee that they do not cut-off the local optimum. NSGBD has been tested on the dynamic optimization of a fluid catalytic cracking unit, but it has not been tested yet in the field of integrated decision-making. GBD has been widely applied in simultaneous design and control problems (e.g., design and control of reactive distillation columns (Vasudevan et al. 2018) and simultaneous design and scheduling problems (e.g., design and scheduling of multipurpose batch plants (X. Lin and Floudas 2001); hence, NSGBD may bring the additional benefit of converging faster to a point that satisfies first order necessary optimality conditions for these problems.

A GBD variant that aims to solve nonconvex MINLP problems to global optimality is nonconvex nested Benders decomposition (NCNBD) (Füllner and Rebennack 2022). This method aims to address nonconvex multistage MINLP problems, where discrete and continuous variables as well as nonlinearities appear in any of the stages. NCNBD combines a variety of tools such as piecewise linear relaxations, regularization, binary approximations, and cutting planes. It relies on the dynamic refinement of the different approximations considered in the method, e.g., piecewise linear Benders cuts are constantly refined as iterations progress. NCNBD involves two iteration loops: in the inner loop, NCNBD optimizes an MIP that outer approximates the original multistage MINLP problem, whereas in the outer loop, NCNBD iteratively refines the outer approximation to improve its accuracy. NCNBD was tested with a variety of unit commitment problems, showing that it becomes more efficient than existing global solvers such as LINDO Global and Couenne as the number of stages increases. As the first decomposition method proposed for general multistage nonconvex MINLPs, NCNBD may be useful to incorporate multistage uncertainty in integrated decision-making MINLP problems, while providing global optimality guarantees. For instance, multistage uncertainty appears in all the operational decision layers (planning, scheduling, and control), which are decision processes executed during multiple sequential instants (i.e., stages) of time, and for which new information is revealed and unforeseen events may occur at any of those decision stages. An illustrative example in this context is the case of optimal generation planning and scheduling in hydro-based power systems over a multiyear planning horizon, which is divided into a series of coupling problems with different time scales including a long-term planning horizon with monthly steps and a shorter scheduling horizon with weekly time steps (Beltrán et al. 2021). Unforeseen events in this case include uncertainty in market prices and inflow energy, which can be modeled and considered by NCNBD as multistage uncertainty.

### 5.4 Column generation

Column generation is traditionally used for large-scale LP and has been embedded within BB in branch-and-price to handle MIP problems. Column generation has been recently extended to solve MINLP problems to global optimality. Allman and Zhang (2021) proposed a branch-and-price method for MINLPs (MINLP-BP), which combines column generation with the global optimization of MINLPs. MINLP-BP relies on the presence of complicating constraints in the formulation, i.e., when complicating constraints are removed, the resulting MINLP problem (or subproblems) can be solved more efficiently. As previously discussed in the Lagrangian decomposition section, integrated decision-making problems in chemical engineering usually follow this structure; however, MINLP-BP has not been used in this field. Nonetheless, MINLP-BP has shown to be useful when solving chemical engineering optimization problems such as the multiscenario synthesis of water networks and the multiscenario design of multiproduct batch plants (Allman and Zhang 2021). Overall, MINLP-BP showed improved performance when compared to BARON, e.g., from 25 problem instances tested, MINLP-BP solved 16 to global optimality, while BARON could not solve any of the instances to global optimality under 10,000 s. Note that this solution strategy exploits problems with complicating constraints; hence, it may be suitable to solve those integrated decision-making problems that have been tackled with LD (e.g., simultaneous refinery planning and crude-oil scheduling), with the additional benefit of providing global optimality guarantees.

Another column generation (CG)-based strategy for MINLPs (MINLP-CG) (Muts et al. 2023) combines CG with the Frank–Wolfe method to generate columns, thus avoiding the need to rely on a BB search. Similarly to DECOA, MINLP-CG also relies on a block-separable reformulation of the MINLP and considers these blocks as columns. Consequently, MINLP-CG is another solution technique that is worth exploring in the context of the integration of two and three decision layers, where each block would represent a decision layer. A key benefit of MINLP-CG is that it enables the implementation of parallel computation strategies to generate new columns, thanks to the block-separable structure of the MINLP. The superstructure-based synthesis of decentralized energy supply systems was used as a case study. When compared to BARON, MINLP-CG showed better dual and primal bounds for problems with thousands of variables, which are often found in energy systems optimization problems.

### 5.5 Logic-based algorithms

Although the first logic-based algorithms appeared in the 1990s, these are becoming popular due to their increasing accessibility. Furthermore, recent research works have developed new logic-based algorithms aimed to facilitate GDP optimization, including integrated decision-making applications. Logic-based solution approaches take advantage of the GDP form of the problem and its underlying logic relationships. The defining characteristic of logic-based algorithms is that they solve the nonlinear subproblems that result from fixing Boolean variables in a reduced space, i.e., nonlinear constraints and variables related to inactive disjunctions are omitted from the subproblem model. This avoids numerical issues (e.g., singularities) and reduces subproblems’ sizes when compared to MINLP solvers. Logic-based solvers that are currently available include logic-based outer approximation (L-OA) (Türkay and Grossmann 1996), global logic-based outer approximation (GL-OA) (Trespalacios and Grossmann 2016), and logic-based branch and bound (L-BB) (Lee and Grossmann 2000). More recently, logic-based D-SDA (LD-SDA) (Bernal et al. 2022a) and logic-based discrete Benders decomposition (LD-BD) (Liñán and Ricardez-Sandoval 2023) have been proposed in the literature, which incorporate discrete convex analysis and logic-based Benders decomposition (LBBD) (Hooker 2019) principles into the direct solution of GDP problems. Similarly to D-SDA, LD-SDA and LD-BD require disjunctions to represent discrete ordered decisions, e.g., choosing an optimal discrete processing time, or selecting an optimal tray in a distillation column.

Despite their benefits, logic-based solvers have not been extensively used yet in the field of integrated MINLP decision-making. To the authors’ knowledge, LD-SDA and LD-BD are the only recent logic-based algorithms that have been presented for integrated decision making. LD-SDA and LD-BD bring together the benefits of logic-based optimization and discrete convex analysis features, e.g., an exploration of disjunctions in reduced-space that relies on neighborhood verification. These algorithms have been tested in the context of simultaneous scheduling and dynamic optimization of network batch plants under variable processing times, showing promising results when compared to general-purpose MINLP solvers (Liñán and Ricardez-Sandoval 2024b), e.g., a multicut LD-BD strategy performed a more efficient exploration of disjuncts, while general-purpose MINLP solvers sometimes stagnated at the initialization. Note that the development of logic-based methods as general-purpose GDP solvers is in an early (developing) stage compared to general-purpose MINLP solvers. Consequently, there are a variety of future research directions, such as using more sophisticated branching techniques in L-BB, or expanding the range of solvable problems with LD-SDA and LD-BD by enabling inclusive and unordered disjunctions in the formulation.

### 5.6 Software tools

The expertise, time, and effort needed for the implementation and coding of MINLP algorithms for integrated decision-making may hinder the adoption of these techniques by engineering practitioners. Although many of the MINLP techniques discussed in Sections 4 and 5 require some degree of customization, many algorithmic steps can be automated and incorporated within new software tools and solvers suitable for integrated decision-making. Some examples of readily available software include the parametric optimization and control (PAROC) framework for the multiparametric optimal design, operation, and control of process systems (Burnak et al. 2020); the bilevel-parametric optimization (B-POP) toolbox that incorporates a variety of solvers for bilevel optimization (Avraamidou and Pistikopoulos 2019); the data-driven optimization of bi-level mixed-integer nonlinear problems (DOMINO) framework that approximates bilevel optimization problems as single level problems through a data-driven approximation of one of the problems levels (Beykal et al. 2020); or the reactive distillation toolbox (RD-toolbox), specifically designed for the integrated design and control of reactive distillation systems (Iftakher et al. 2022). Furthermore, automated tools for chemical process flowsheet synthesis that can deal with GDP problems using advanced machine learning frameworks are emerging in the literature (Mann et al. 2024; Reynoso-Donzelli and Ricardez-Sandoval 2024).

In the context of recent MINLP algorithmic developments, the mixed-integer nonlinear decomposition toolbox in Pyomo (MindtPy) incorporates traditional MINLP techniques based on BB and OA, along with recently proposed strategies such as ROA and a feasibility pump for OA (Bernal et al. 2018). GDP modeling and logic-based algorithms are also becoming more accessible to the general public, thanks to recently developed packages for algebraic modeling languages (Chen et al. 2022; Perez et al. 2023). Particularly, the GDPopt solver in Pyomo not only enables GDP modeling and MINLP transformations (e.g., Big-M) but also provides access to logic-based solvers such as LOA, GLOA, and LBB (Chen et al. 2022).

## 6 Summary and outlook

A variety of advanced solution strategies have been proposed in the literature to address integrated decision-making MINLP problems in chemical engineering such as reformulations (e.g., NLP reformulations), variable decomposition techniques (e.g., Benders-based strategies), and constraint decomposition approaches (e.g., Lagrangian decomposition strategies). These techniques avoid the issues that arise with the direct application of general-purpose MINLP solvers, e.g., failure in the presence of highly nonlinear problems leading to feasible problems erroneously detected as infeasible, or lengthy computational times that do not comply with fast-paced production environments. While the advanced integrated decision-making strategies reviewed in this work are often applied to integrate two decision layers (e.g., scheduling and control or process design and control), the simultaneous consideration of three or more decision layers is seldom considered in the literature. From an optimization viewpoint, the integration of three or more decision layers is not only relevant when the corresponding objective functions include terms from the different layers but also when the linking variables that interconnect these layers act as interacting factors that cannot be treated independently, i.e., the problem cannot be decomposed into multiple independent subproblems. A notable example is the optimal MINLP integration of planning, scheduling, and control, for which several studies have been identified in the literature; most of them dealing with a single multiproduct unit operation (Charitopoulos et al. 2019; Gutiérrez-Limón et al. 2014; Mora-Mariano et al. 2020). For this integrated problem, and under a profit maximization objective, optimal integration may be needed when frequent and economic transitions between multiple products are needed (i.e., the process rarely operates at steady state); the production forecast is driven by a scheduled production goal whose cost is given by the production order and transition costs, and the planned production goals that affect the scheduling decisions are optimized based on constantly changing supply and demand requirements. Another research area that should be explored further is the development of software tools for both MINLP and GDP integrated decision-making. For instance, software particularly tailored for MINLP integrated decision-making through mixed integer or continuous reformulations, Lagrangian relaxation, or Back-Off algorithms are not available in the literature.

To fill in the existing literature gaps, recent advances from the broader field of deterministic MINLP and GDP algorithms can be taken into consideration for the development of more advanced and efficient integrated decision-making techniques and software tools. Local solution techniques can be proposed in the future for problems involving feasibility challenges and block-separable structures, through regularization, feasibility-path optimization, and improved convergence guarantees to local optima. Similarly, global optimization techniques that rely on column generation, branch-and-price, and nonconvex Benders decomposition can be further explored to solve existing integrated decision-making problems to global optimality. In terms of applications, the interconnection between decision layers in the field of MINLP is often investigated for traditional unit operations (e.g., CSTR reactors, ideal-binary distillation systems) but scarcely explored for more challenging processes such as process intensification technologies, integrated energy systems, or bioprocesses. These systems may be highly nonlinear, and their optimization models are difficult to converge; thus, the development of efficient MINLP and GDP optimization techniques based on the contributions summarized in this work may foster future research in this area.
